# Golgi Protein 73 (GP73) Serum Levels Predict Outcome after Resection of Biliary Tract Cancer

**DOI:** 10.3390/cancers14184428

**Published:** 2022-09-12

**Authors:** Sven H. Loosen, Justus Halpaap, Simon Labuhn, Jan Bednarsch, Patrick H. Alizai, Anjali A. Roeth, Sven A. Lang, Mihael Vucur, Jakob N. Kather, Wolfram T. Knoefel, Tom F. Ulmer, Ulf P. Neumann, Christoph Roderburg, Tom Luedde

**Affiliations:** 1 Clinic for Gastroenterology, Hepatology and Infectious Diseases, University Hospital Düsseldorf, Medical Faculty of Heinrich Heine University Düsseldorf, 40225 Düsseldorf, Germany; 2 Department of Medicine III, University Hospital RWTH Aachen, Pauwelsstrasse 30, 52074 Aachen, Germany; 3 Department of Visceral and Transplantation Surgery, University Hospital RWTH Aachen, Pauwelsstrasse 30, 52074 Aachen, Germany; 4 Else Kroener Fresenius Center for Digital Health, Medical Faculty Carl Gustav Carus, Technical University Dresden, 01069 Dresden, Germany; 5 Department of General, Visceral and Pediatric Surgery, University Hospital Düsseldorf, Medical Faculty of Heinrich Heine University Düsseldorf, 40225 Düsseldorf, Germany

**Keywords:** BTC, cholangiocarcinoma, CCA, prognosis, biomarker, surgery

## Abstract

**Simple Summary:**

Biliary tract cancer (BTC) represents a rare liver malignancy with unfavorable outcome. It is often challenging to identify the ideal surgical candidates and present stratification algorithms are rarely based on the individual tumor biology. In the present manuscript, we evaluated a role of serum Golgi protein 73 (GP73) in patients with resectable BTC. We could show that elevated levels of GP73 before surgery identified a subgroup of BTC patients with a significantly reduced overall survival after tumor resection. Therefore, measurement of GP73 serum levels might become a novel tool in the challenging preoperative stratification process of patients with resectable BTC.

**Abstract:**

Background: Tumor resection represents the only potentially curative therapy for patients with biliary tract cancer. Nevertheless, disease recurrence is observed in about 50% of patients, leading to a 5-years survival rate of less than 50%. The Golgi protein 73 (GP73), a type II Golgi transmembrane protein, exerts important functions of intracellular protein processing and transportation. Circulating GP73 has recently been suggested as a prognostic marker following resection of hepatocellular carcinoma (HCC) but its role in the context of BTC has remained unknown. In this study, we evaluate a potential role of circulating GP73 as a novel biomarker in patients with resectable BTC. Methods: GP73 serum levels were measured by immunoassay in n = 97 BTC and n = 40 HCC patients as well as n = 31 healthy controls. Results were correlated with clinical data. Results: Serum GP73 levels were significantly elevated in BTC patients compared to healthy controls but lower compared to HCC patients. The combination of GP73/CA19-9 showed a sensitivity and specificity of 83.5% and 90.3% regarding the differentiation of BTC patients and healthy controls. BTC patients with baseline GP73 levels above the ideal cut-off value (42.47 ng/mL) showed a significantly reduced median overall survival (193 days) compared to patients with preoperative GP73 levels below this cut-off (882 days). These results were confirmed in uni- and multivariate Cox-regression analysis including several clinicopathological parameters such as age, ECOG performance status, tumor stage as well as established tumor markers and parameters of liver and kidney function. Conclusions: GP73 represents a previously unrecognized biomarker in the patients with resectable BTC that identifies patients with an impaired postoperative outcome. If larger clinical trials confirmed these findings, measurement of GP73 serum levels might become a novel tool in the challenging preoperative stratification process of patients with resectable BTC.

## 1. Introduction

Incidence rates of biliary tract cancer (BTC) are rising globally [1,2]. Surgical tumor resection in combination with adjuvant systemic therapy is the only potentially curative therapy for individual early-stage patients [1,2] but disease recurrence occurs in about 60% of successfully resected patients [3]. The 5-years survival rates of recent clinical trials investigating different adjuvant therapy regimens have remained below 50% [3]. Importantly, tumor resection is only feasible if BTC is diagnosed at an early disease stage and also in these patients, prediction of postoperative outcome in terms of disease recurrence represents a major challenge. In this context, it is important to note that the decision regarding tumor resection or alternative therapeutic approaches such as chemotherapy is currently mainly based on surgical resectability and clinical aspects [4].

The Golgi protein 73 (GP73) also known as Golgi membrane protein 1 (GOLM1) is a Golgi type II transmembrane protein that exerts decisive intracellular functions such as the processing and transportation of proteins through the Golgi apparatus and was first described in the context of viral hepatitis [5]. In the context of hepatocellular carcinoma (HCC), both tissue expression and circulating levels of GP73 are upregulated [6], and GP73 has been associated with pro-malignant characteristics such as proliferation, migration, and metastasis [7,8]. Serum levels of GP73 have moreover been suggested as a diagnostic marker for early detection of HCC [9] and elevated GP73 levels predicted an unfavorable following hepatectomy in HCC patients [10]. In contrast, only very little is known about the potential function of GP73 in the context of BTC. Here, we therefore aimed at investigating a potential diagnostic and/or prognostic role of GP73 serum levels in n = 97 BTC patients who received tumor resection at out tertiary referral center between 2011 and 2017.

## 2. Patients and Methods

### 2.1. Study Design and Patient Cohort

This observational cohort study aimed at evaluating circulating GP73 serum levels as a potential novel diagnostic or prognostic biomarker in patients with resectable BTC. We included a total of n = 97 BTC patients who were admitted for tumor resection to the Department of Visceral and Transplantation Surgery at University Hospital RWTH Aachen between 2011 and 2017 (Table 1 provides a detailed overview on patient characteristics; the laboratory parameters of the study cohort are shown in Supplementary Table S1). Blood samples were drawn shortly before tumor resection and about one week after (6 or 7 days) for cancer patients or before during blood donation for healthy controls (S-Monovette^®^ Serum-Gel, brown, 7.5 mL, Sarstedt AG & Co. KG, Nümbrecht, Germany) and centrifuged at 2000× *g* for 10 min. Serum samples were then stored at −80 °C until GP73 measurements. BTC was histologically confirmed after tumor resection. We also included n = 31 healthy and cancer-free blood donors as well as n = 40 HCC patients as the control population. Clinical follow-up data were obtained either directly from the clinic’s internal documents, by consulting the patient’s general practitioners, or by querying the death register of the registry office. Patients without available proof of death at the time of data analysis were censored at the time of the last physician contact (clinic or general practitioner). The study protocol was approved by the ethics committee of the University Hospital RWTH Aachen, Germany (EK 206/09) and conducted in accordance with the ethical standards laid down in the Declaration of Helsinki. Written informed consent was obtained from all patients.

### 2.2. Measurements of GP73

GP73 serum levels were measured by enzyme-linked immunosorbent assay (ELISA) according to the manufacturer’s instructions (Human GOLM1/GP-73 ELISA, ELH-GOLM1-1, RayBiotech Life, Inc. Peachtree Corners, GA 30092, USA). Standard laboratory parameters were measured by the common laboratory facility at University Hospital RWTH Aachen.

### 2.3. Statistical Analysis

Shapiro-Wilk test was performed to test for normal distribution. Comparison of non-parametric was performed using the Mann–Whitney U test or the Kruskal–Wallis test in case of multiple groups. Wilcoxon signed-rank test was applied to compare related samples (e.g., pre- and post-OP). Spearman’s correlation coefficient was used for correlation analyses. The optimal diagnostic cut-off was calculated using the Youden-Index method. Kaplan–Meier estimates display the impact of GP73 on overall survival. Log-rank test was used for comparison between the subgroups. The ideal prognostic cut-off value was calculated by fitting Cox proportional hazard models to the dichotomized survival status and the survival time and defining the optimal cut-off as the point with the most significant split in the log-rank test using RStudio (version 1.2.5033 (RStudio Inc., Boston, MA, USA). The prognostic value of GP73 was also analyzed in uni- and multivariate Cox regression analyses. Parameters showing a *p*-value < 0.150 in univariate analysis were included into multivariate analysis. The hazard ratio (HR) and the 95% confidence interval are shown. All statistical analyses were performed with SPSS 23 (SPSS, Chicago, IL, USA) [11]. A *p* < 0.05 was considered statistically significant (* *p* < 0.05; ** *p* < 0.01; *** *p* < 0.001).

## 3. Results

### 3.1. Patient Characteristics

The biliary tract cancer (BTC) cohort included n = 97 patients, of which 46.9% were female and 53.1% were male. The median age was 68 years and ranged from 37 to 84 years. Intrahepatic cholangiocarcinoma (CCA) (39.2%) and perihilar tumors (38.1%) represented the most common tumor entities, while distal CCA (13.4%) and gallbladder cancer (9.3%) were less frequent. A total of 50.5% of patients presented with a good ECOG performance status, whereas 40.0% (ECOG 1) and 9.5% (ECOG 2) showed an impaired performance status upon initial presentation. Detailed characteristics of the study cohort are displayed in Table 1.

### 3.2. Golgi Protein 73 Serum Levels Are Elevated in Patients with Biliary Tract Cancer

**Figure 1:**
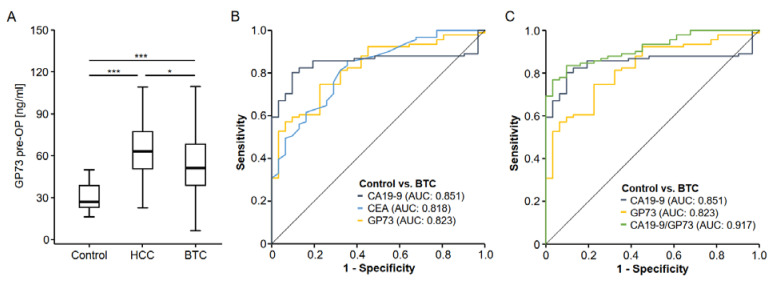
Circulating levels of GP73 in HCC and BTC patients. (**A**) Patients with hepatocellular carcinoma (HCC) and biliary tract cancer (BTC) both have significantly increased GP73 serum levels compared to healthy controls. The induction of circulating GP73 is significantly stronger in HCC compared to BTC. * *p* < 0.05; *** *p* < 0.001. (**B**) Circulating GP73 levels show a slightly lower AUC value than CA19-9 but a similar AUC value compared to CEA serum levels. (**C**) The combination of GP73 and CA19-9 serum levels has the AUC value for the identification of BTC cancer patients.

Before investigating the role of GP73 in the serum of BTC patients, we first aimed at confirming existing data on an upregulation of circulating GP73 in patients with hepatocellular carcinoma (HCC). Compared to healthy control samples (n = 31), HCC patients (n = 40) showed a significant 2.34-fold elevation of GP73 serum values (Figure 1A). We then analyzed GP73 serum levels in BTC patients and also observed significantly increased levels compared to healthy controls (Figure 1A). The induction of circulating GP73 in BTC (2.01-fold) was however significantly lower compared to HCC patients (Figure 1A). To evaluate the diagnostic potential of GP73 in comparison to clinically approved tumor markers such as CEA and CA19-9, we performed ROC curve analysis, which revealed an AUC value of 0.823 for GP73 regarding the differentiation between BTC patients and healthy controls (Figure 1B). Using the ideal diagnostic cut-off value (38.88 ng/mL), the diagnostic sensitivity and specificity of circulating GP73 was 75.3% and 77.4%, respectively. The AUC value of GP73 was slightly lower compared to CA19-9 (AUC_CA19-9_: 0.851) but comparable to CEA (AUC_CEA_: 0.818, Figure 1B). When looking at the combination of GP73 and CA19-9, the diagnostic potential further increased compared to either marker alone, showing an AUC value of 0.917 (Figure 1C). For the combination of GP73 and CA19-9, the diagnostic sensitivity and specificity were 83.5% and 90.3%.

### 3.3. Golgi Protein 73 Serum Levels Are Unaltered between Different Tumor and Patient Characteristics

**Figure 2:**
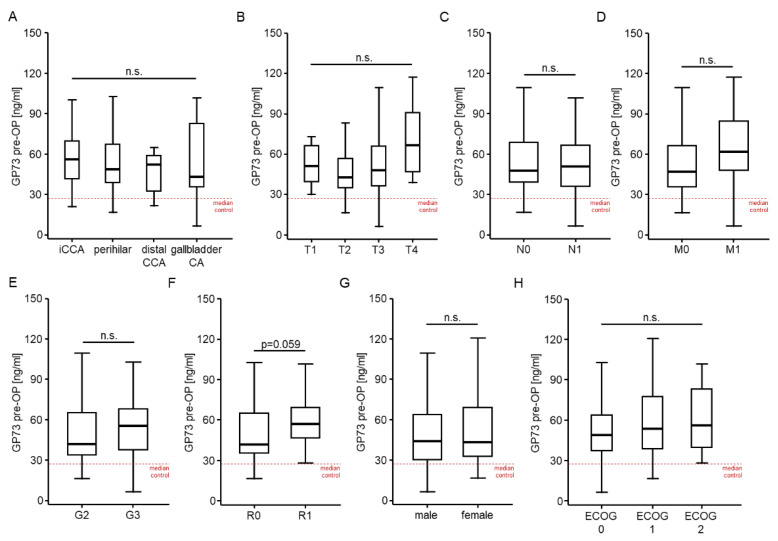
GP 73 serum levels are unaltered between different tumor and patient characteristics. Initial GP73 serum levels are unaltered between patients with different tumor localizations (**A**), different TNM-stages (**B**–**D**), and tumor grading (**E**). (**F**) Patients who were resected with microscopically remaining tumor cells (R1) show a trend towards higher baseline GP73 levels, though statistical significance is not reached. Serum GP73 levels are unaltered between male and female patients (**G**) as well as patients with different ECOG performance status (**H**).

To gain further knowledge into the potential drives of elevated GP73 levels in patients with BTC, we next compared GP73 levels between patients with different disease characteristics. However, GP73 serum levels were unaltered between patients with different tumor localization (intrahepatic CCA, perihilar tumors, distal CCA, and gallbladder cancer, Figure 2A), different TNM-status (Figure 2B–D), or different tumor grading (moderately differentiated (G2) vs. poorly differentiated (G3), Figure 2E). Patients who were resected with microscopically remaining tumor cells (R1) showed a trend towards higher baseline GP73 levels, though statistical significance was not reached (*p* = 0.059, Figure 2F). Finally, serum GP73 levels were unaltered between male and female patients (Figure 2G) as well as patients with different ECOG performance status (Figure 2H).

Correlation analysis between baseline GP73 levels and standard laboratory markers of organ dysfunction as well as BTC tumor markers revealed a significant positive correlation between baseline GP73 levels and CEA (r_S_:0.299, *p* = 0.004) as well as CA19-9 levels (r_S_:0.406, *p* < 0.001). While GP73 levels did not correlate with serum markers of liver (AST, bilirubin, ALP and GGT) or renal dysfunction (creatinine), we observed a significant positive correlation between GP73 and CRP serum levels (r_S_:0.393, *p* < 0.001, Supplementary Table S2).

### 3.4. Elevated Baseline Levels of GP73 Predict an Impaired Overall Survival following BTC Resection

**Figure 3:**
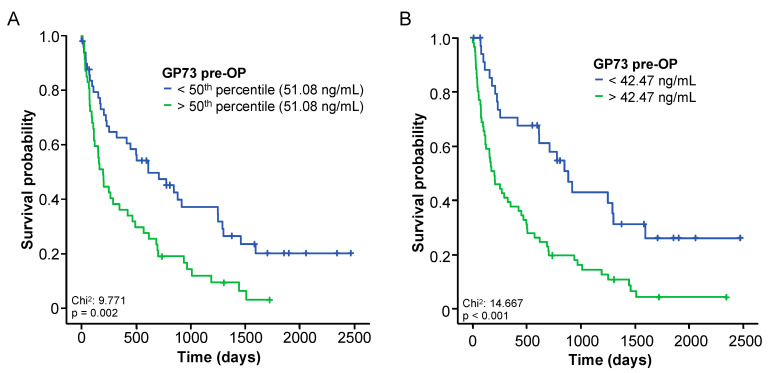
Elevated baseline levels of GP73 predict an impaired overall survival following BTC resection. (**A**) BTC patients with baseline GP73 serum levels above the 50th percentile (51.08 ng/mL) have a significantly impaired postoperative outcome. (**B**) At the optimal prognostic cut-off value (42.47 ng/mL), baseline GP73 levels identify a subgroup of BTC patients with a significantly impaired overall survival after tumor resection.

Based on the strong induction of circulating GP73 in BTC patients, we subsequently hypothesized that elevated GP73 levels might be associated with an impaired outcome after tumor resection. We therefore first compared the overall survival (OS) of BTC patients with initial GP73 serum levels above or below the median GP73 level of 51.08 ng/mL. In this analysis, patients with baseline GP73 levels > 51.08 ng/mL showed a significantly reduced OS compared to patients with GP73 levels < 51.08 ng/mL (*p* = 0.002, Figure 3A). To further increase the prognostic potential of circulating GP73, we then calculated an optimal cut-off value of 42.47 ng/mL, which differentiated best between patients with a good or poor outcome. When using this cut-off value, Kaplan–Meier estimates showed a highly reduced OS for BTC with baseline GP73 levels above 42.47 ng/mL (*p* < 0.001, Figure 3B). The median OS was 193 days in the “GP73-high” (>42.47 ng/mL) and 882 days in the “GP73-low” group (<42.47 ng/mL).

The prognostic potential of baseline GP73 levels was further confirmed in the Cox-regression model. In univariate analysis, initial GP73 levels above 42.47 ng/mL were a significant prognostic factor for OS (HR: 2.559, 95% CI: 1.556–4.209, *p* < 0.001). In addition, we identified several clinical parameters (age, ECOG PS, BMI, tumor stage) as well as different laboratory parameters (CEA, CRP, and creatinine levels) as prognostic as potentially prognostic factors (*p* < 0.150) in univariate Cox-regression analyses (Table 2). Importantly, initial GP73 serum levels proved to be independent of these confounders in multivariate Cox-regression analysis (HR: 2.457, 95% CI: 1.255–4.808, *p* = 0.009, Table 2).

### 3.5. Postoperative GP73 Serum Levels and Patients’ Outcome

**Figure 4:**
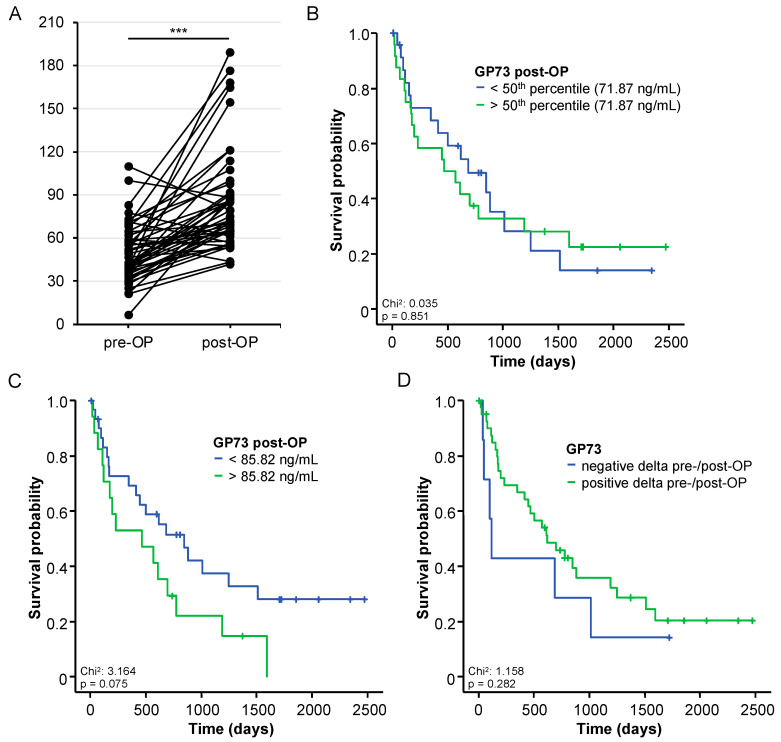
Postoperative GP73 levels and patients’ outcome. (**A**) Circulating levels of GP73 are significantly elevated at days 6 or 7 following tumor resection. *** *p* < 0.001. (**B**) Postoperative GP73 levels above the 50th percentile (51.08 ng/mL) are unsuitable to predict postoperative outcome. (**C**) BTC patients with postoperative GP73 levels above >85.82 ng/mL show a strong trend towards an impaired overall survival, though statistical significance is not reached. (**D**) The overall survival is not significantly altered between patients with increasing or decreasing GP73 levels after tumor resection.

Postoperative serum levels were taken 6 or 7 days after tumor resection and were available for n = 48 BTC patients. Compared to the patients’ respective preoperative level (median: 51.08 ng/mL), GP73 serum levels showed a significant increase following tumor resection (median: 71.87 ng/mL, *p* < 0.001, Figure 4A). With respect to the postoperative outcome, we did not observe a significant difference of overall survival (OS) between patients with postoperative GP73 levels above or below the median GP73 level of 71.87 ng/mL (*p* = 0.851, Figure 4B). Again, we calculated an ideal prognostic cut-off value for postoperative GP73 levels (85.82 ng/mL). Although patients with postoperative GP73 levels above >85.82 ng/mL showed a strong trend towards an impaired OS, statistical significance was not reached (*p* = 0.075, Figure 4C). Moreover, OS was not significantly altered between patients with increasing or decreasing GP73 levels after tumor resection (*p* = 0.282, Figure 4D). Finally, we aimed at further dissecting the underlying mechanism driving the GP73 elevation after tumor resection. Hypothesizing that postoperative inflammation might represent a key factor involved in the observed upregulation, we correlated postoperative GP73 levels with postoperative CRP levels as a surrogate of systemic inflammation. Here, we observed a strong positive correlation (r_S_:0.412, *p* = 0.005), suggesting that inflammation is indeed a potential driver of elevated postoperative GP73 levels.

## 4. Discussion

The prognosis of biliary tract cancer (BTC) has remained very poor. Surgical tumor resection, which can potentially lead to a cure for individual patients, is only feasible if patients are diagnosed at an early disease stage. Moreover, the majority of resected patients eventually develop disease recurrence, resulting in a palliative therapeutic approach with dismal outcome [1,2]. Importantly, the preoperative identification of patients with an unfavorable postoperative outcome has remained challenging and the decision for or against tumor surgery is often based on clinical factors and the surgical resectability only [12]. Together, novel biomarkers facilitating an early diagnosis of BTC on the one hand and allowing a reliable prediction of the patients’ postoperative outcome on the other are urgently needed to improve the outcome of BTC patients. To the best of our knowledge, our study is the first to describe a potential prognostic role of circulating GP73 in the context of BTC. We showed that GP73 serum levels are significantly elevated in BTC patients compared to healthy controls and further increased the diagnostic power of established tumor markers such as CA19-9. Moreover, we identified a significant role of baseline GP73 levels as a prognostic marker of overall survival (OS) following tumor resection. As such, patients with preoperative GP73 serum levels above the established ideal cut-off value of 42.47 ng/mL showed a significantly reduced median OS of 193 days compared to 882 days in patients with baseline GP73 serum levels below the cut-off value. These results were confirmed by uni- and multivariate Cox-regression analysis including several clinicopathological parameters such as age, tumor stage, ECOG performance status, and established BTC tumor markers.

GP73 represents a Golgi transmembrane protein that exerts important intracellular functions such as the processing and transportation of proteins in the Golgi apparatus and was first described in the context of viral hepatitis in the year 2000 [5]. Overexpression of GP73 has also been reported in activated hepatic stellate cells promoting of liver fibrosis and chronic liver disease [13]. In the context of hepatocellular carcinoma (HCC), both tissue expression and circulating levels of GP73 are upregulated [6], and GP73 has been associated with pro-malignant characteristics such as proliferation, migration, and metastasis [7,8]. On a molecular level, GP73 was shown to selectively interact with the epidermal growth factor receptor (EGFR) and function as a cargo adaptor to support EGFR/RTK anchoring on the trans-Golgi network (TGN) and the recycling back to the cell membrane, thereby prolonging the activation of downstream targets [7]. GP73 also enhances p-Smad2 and p-Smad3 by TGF-β1, resulting in the promotion of Epithelial-Mesenchymal Transition and invasion of HCC cells [14]. GP73 expression itself was shown to be upregulated by mTORC1 and promotes growth in an HCC xenograft mouse model [8]. Serum levels of GP73 have also been suggested as a diagnostic marker for early HCC detection [9] as well as a prognostic marker following hepatectomy in patients with HCC [10]. In line, we confirmed a significant upregulation of serum GP73 levels in a smaller cohort of n = 40 HCC patients. In contrast, data on a potential role of GP73 in BTC are limited. Strong expression of GP73 was shown in 97/114 BTC tissue samples and proved as an independent prognostic factor for overall survival [6]. Our data support the hypothesis that the tumoral overexpression of GP73 is reflected by elevated GP73 serum levels in BTC patients. In line with tissue expression levels, circulating levels of GP73 were also an independent predictor of survival in our cohort. In this line of thinking, it is important to note that circulating GP73 levels represent an easily accessible biomarker that does not require invasive tumor biopsy and might thus be the preferable tool in clinical routing. Our study does however not provide information on a pathophysiological role linking elevated preoperative GP73 levels with an impaired postoperative outcome. Thus, further studies investigating the molecular function by, e.g., GP73 knockdown experiments [15], are warranted to further dissect a potentially functional role of GP73 in BTC.

The clinical implementation of a novel biomarker into daily routine is challenging. Although several individual diagnostic and/or prognostic biomarkers for BTC have been suggested previously [16], almost none of them are used in clinical routine mainly because these markers are not disease-specific and also elevated in non-malignant diseases. In line, GP73 serum levels showed a good performance in differentiating between BTC and healthy controls, but were also elevated in HCC patients. In this context, we suggest that GP73 serum levels should be implemented into, e.g., diagnostic algorithms including several parameters rather than being used as a single marker. This suggestion is supported by the fact that in our cohort the combination of CA19-9 and GP73 showed a diagnostic potential compared to either marker alone. In this context, it should be noted that patients with the Lewis phenotype (a-b-) do not have elevated CA19-9 levels and are therefore often falsely diagnosed as negative [17]. In similar approach, the combination of GP73, glypican-3, and Alpha-Fetoprotein outperformed all other biomarkers in a large meta-analysis including 919 HCC patients [18]. A clinical implementation of the prognostic role of baseline GP73 levels would also warrant a more detailed consideration. Although our study does not provide information on the important question whether or not a BTC patients with high initial GP73 levels might have benefitted to a greater extend from alternative treatment option, it is unlikely that a patient with resectable BTC will be denied surgery due to GP73 levels above our ideal cut-off value. As highly active chemotherapy regimens are evaluated in clinical trials (NCT02170090), high GP73 before tumor resection could also trigger a more aggressive line of adjuvant therapy for individual BTC patients.

When analyzing postoperative GP73 serum levels at one week after tumor resection, we observed significantly higher levels compared to the baseline levels. This finding argues against the hypothesis that BTC cells represented the only source of circulating GP73 in our cohort of patients. It is likely that circulating GP73 also originates, e.g., from the tumor microenvironment and other populations of liver-resident cells such as hepatic stellate cells, which have recently been described as a source of GP73 [13]. Moreover, postoperative systemic inflammation might trigger GP73 elevation after tumor resection. Interleukin-6 (IL-6), a pro-inflammatory cytokine, has been shown to upregulate GP73 mRNA and protein levels in HepG2 cells and GP73 serum levels correlated with IL-6 levels in patients with pre-malignant liver disease [19]. In this line of thinking, we observed a significant correlation between postoperative GP73 and CRP serum levels, suggesting that postoperative inflammation indeed represents a key driver of elevated postoperative GP73 levels. Thus, further studies evaluating GP73 levels at later postoperative time points are warranted to fully elucidate the longitudinal course of GP73 following BTC tumor resection.

Our study was limited by some points. First, our analysis features a single-center design arguing that the results need to be confirmed in multicenter studies using independent BTC patient cohorts before an implementation of GP73 in clinical routine could be considered. Second, we only included patients undergoing resection of BTC and are thus unable to compare the outcome of patients with high or low GP73 serum levels who received a different therapeutic approach such as systemic chemotherapy. Third, there was no matching of healthy control samples for patients’ age or sex.

## 5. Conclusions

In summary, our data suggest a diagnostic and/or prognostic role of circulating GP73 in patients with resectable BTC. If confirmed in larger clinical trials, ideally including other non-malignant inflammatory biliary conditions such as primary sclerosing cholangitis (PSC), these findings argue for a potential clinical implementation of GP73 in both diagnostic algorithms as well as preoperative stratification strategies to improve the individual outcome of BTC patients in future.

## Figures and Tables

**Table 1:** Characteristics of the study population.

	BTC Patients	Healthy Controls	*p*-Value
BTC patients	n = 97	n = 31	
Gender [%]:			
Male	53.1	76.7	0.033
Female	46.9	23.3	
Age [years, median and range]	68 [37–84]	33 [19–74]	<0.001
BMI [kg/m^2^, median and range]	26.23 [18.83–46.36]		
Anatomic location of BTC [%]:			
Intrahepatic CCA	39.2		
Perihilar	38.1		
Distal CCA	13.4		
Gallbladder	9.3		
Staging [%]:			
T1-T2-T3-T4	6.3-41.3-35.0-17.5		
N0-N1	43.2-56.8		
M0-M1	77.9-22.1		
G2-G3	53.7-46.3		
R0-R1	60.3-39.7		
ECOG PS [%]:			
ECOG 0	50.5		
ECOG 1	40.0		
ECOG 2	9.5		
Deceased during follow up [%]:			
Yes	81.4		
No	18.6		

BTC: biliary tract cancer, CCA: cholangiocarcinoma, BMI: body mass index, ECOG PS: “Eastern Cooperative Oncology Group” performance status.

**Table 2:** Uni- and multivariate Cox-regression analyses regarding overall survival.

	Univariate Cox-Regression		Multivariate Cox-Regression	
Parameter	*p*-Value	Hazard-Ratio (95% CI)	*p*-Value	Hazard-Ratio (95% CI)
GP73 pre-OP >42.47 ng/mL	<0.001	2.559 (1.556–4.209)	0.009	2.457 (1.255–4.808)
CEA	0.059	1.004 (1.000–1.008)	0.006	1.009 (1.002–1.015)
Leukocyte count	0.451	1.024 (0.962–1.091)		
CRP	<0.001	1.012 (1.006–1.018)	0.469	1.003 (0.994–1.013)
Platelets	0.902	1.000 (0.999–1.002)		
Sodium	0.210	0.959 (0.889–1.024)		
Potassium	0.904	1.029 (0.641–1.653)		
AST	0.407	0.999 (0.998–1.001)		
Bilirubin	0.325	0.967 (0.903–1.034)		
ALP	0.797	1.000 (0.999–1.001)		
GGT	0.514	1.000 (0.999–1.000)		
Creatinine	0.104	1.918 (0.874–4.208)	0.328	0.588 (0.202–1.706)
BMI	0.061	1.048 (0.998–1.100)	0.026	1.070 (1.008–1.136)
ECOG PS	0.085	1.371 (0.957–1.964)	0.108	1.500 (0.915–2.461)
Age	0.014	1.027 (1.005–1.050)	0.058	1.029 (0.999–1.060)
Sex	0.984	1.005 (0.643–1.579)		
T-stage	0.004	1.633 (1.171–2.278)	0.051	1.461 (0.999–2.136)

GP73: golgi protein 73, CEA: carcinoembryonic antigen, CRP: C-reactive protein, AST: aspartate transaminase, ALP: alkaline phosphatase, GGT: Gamma-glutamyltransferase, BMI: Body-Mass-Index, ECOG PS: “Eastern Cooperative Oncology Group” performance status.

## Data Availability

The data are available upon reasonable request from the corresponding author.

## Supplementary Materials

The following supporting information can be downloaded at: www.mdpi.com/article/10.3390/cancers14184428/s1, Table S1: Various laboratory parameter of study cohort (median, range). Table S2: Correlation analysis between baseline GP73 and tumor markers as well as markers of organ dysfunction.
