# Supplementary material for: Golgi Protein 73 (GP73) Serum Levels Predict Outcome after Resection of Biliary Tract Cancer
Source: Cancers (Basel). 2022 Sep 12;14(18):4428. doi: 10.3390/cancers14184428 (PMC9497317; doi:10.3390/cancers14184428)
Supplement: Supplementary file 1 [file cancers-14-04428-s001.zip › cancers-1838578-supplementary.pdf]

Supplementary Table S1. Various laboratory parameter of study cohort (median, range).

**Laboratory parameter**	**BTC patients** (n=97)	**Healthy controls** (n=31)
GP73 pre-OP [ng/ml]	51.08 [6.45-156.94]	26.97 [16.26-64.80]
GP73 post-OP [ng/ml]	71.87 [41.65-188.94]	-
CEA [µg/l]	3.0 [0.71-333.0]	1.30 [0.30-4.30]
CA 19-9 [U/ml]	79.4 [0.6-22911.0]	5.70 [0.0-44.10]
Leukocyte count [cells/nl]	7.85 [2.9-21.6]	
CRP [mg/l]	18.0 [0.0-230.0]	
Platelets [cells/nl]	266.5 [65.0-931.0]	
Hemoglobin [g/l]	12.45 [7.8-17.1]	
Sodium [mmol/l]	140.0 [131.0-146.0]	
Potassium [mmol/l]	4.4 [2.9-6.1]	
AST [U/l]	45.5 [18.0-1587.0]	28.0 [20.0-78.0]
Bilirubin [mg/dl]	1.0 [0.24-21.49]	0.42 [0.1-0.92]
ALP [U/l]	219.5 [53.0-1055.0]	68.0 [40.0-98.0]
GGT [U/l]	311.0 [36.0-1794.0]	18.0 [8.0-98.0]
Creatinine [mg/dl]	0.9 [0.43-2.08]	

BTC: biliary tract cancer, GP73: golgi protein 73, CEA: carcinoembryonic antigen, CA 19-9: carbohydrate-Antigen 19-9, CRP: C-reactive protein, AST: aspartate transaminase, ALP: alkaline phosphatase, GGT: gamma-glutamyltransferase

Supplementary Table S2. Correlation analysis between baseline GP73 and tumor markers as well as markers of organ dysfunction.

**Parameter**	**GP73**	
	**r_S**	**p-value**
CEA	0.299	0.004
CA19-9	0.406	<0.001
Leukocyte count	0.105	0.311
CRP	0.393	< 0.001
Platelets	-0.086	0.407
Creatinine	0.122	0.235
Sodium	-0.169	0.055
Potassium	-0.041	0.688
AST	0.091	0.378
Bilirubin	0.142	0.169
ALP	0.131	0.208
GGT	0.047	0.655

GP73: golgi protein 73, CEA: carcinoembryonic antigen, CA 19-9: carbohydrate-Antigen 19-9, CRP: C-reactive protein, AST: aspartate transaminase, ALP: alkaline phosphatase, GGT: gamma-glutamyltransferase
